# Transcriptomic perspectives of memory-like NK cells and aging

**DOI:** 10.1186/s13073-022-01059-1

**Published:** 2022-05-25

**Authors:** Dandan Wang, Subramaniam Malarkannan

**Affiliations:** 1 Blood Research Institute, Versiti Inc, 8727 Watertown Plank Rd, Milwaukee, WI 53226 USA; 2 Department of Microbiology and Immunology, Medical College of Wisconsin (MCW), Milwaukee, WI 53226 USA; 3 Division of Hematology and Oncology, Department of Medicine, MCW, Milwaukee, WI 53226 USA; 4 Division of Hematology, Oncology, and Bone Marrow Transplantation, Department of Pediatrics, MCW, Milwaukee, WI 53226 USA

**Keywords:** Memory-like NK cells, Aging, Inflammaging, HCMV, COVID-19

## Abstract

A recent study highlights the presence of a unique memory-like natural killer (NK) cell subset, which accumulates with aging and appears to associate withdisease severity in COVID-19 patients. While the clinical relevance of memory in NK cells is being debated, the molecular identity of this subset in the form of a single-cell transcriptome is essential to define their origin, longevity, functions, and disease relevance.

## Aging and the immune system

Senescence and inflammation are two critical components associated with aging. One mechanism that causes irreversible replicative arrest in cells is the constant loss of telomeric DNA due to the 5′-3′ directionality of DNA polymerase and the inability of the replication machinery to reach the end of chromosomes [1]. However, the confounding multicellular interplay at the systemic level possesses unique challenges. Cellular senescence is a hallmark of aging, and the immune system plays an essential role in both causing and eliminating dying cells. Multiple attempts have been made to determine the coordinated global transcriptomic changes at the organismal level that might explain the molecular basis of aging [2]. Immune cells also undergo increased senescence with aging, a process termed, immune senescence. A low-level persistent inflammation at an older age is defined as inflammaging, a poorly understood aging process. These factors compound the risk factors for seasonal viral infections, malignancies, and chronic inflammatory diseases. A decrease in the clonotypic diversity of T and B cells, the two pillars of adaptive immunity, also demonstrates the diminishing memory formation complicating vaccination modalities for the aged population. However, aging-realted changes in NK cells, the major innate lymphocyte subset, are not fully understood.

In this context, Guo et al. discovered a memory-like proinflammatory CD52^+^NKG2C^+^CD94^+^ NK subset accumulating with aging using single-cell transcriptome [3]. Notably, the accumulation of the CD52^+^NKG2C^+^CD94^+^ NK subset correlated with the severity of COVID-19 pathology. The predominant Type-I interferons (IFN-α/β) increase with aging; however, its role on NK cells in the elderly is not fully understood. The authors find that the memory-like NK cells exhibit a single-cell transcriptomic profile indicative of their ongoing response to type-I interferons. These findings raise several questions. First, do memory-like NK cells contribute to inflammaging in the elderly? Are these memory-like NK cells the predominant contributors to the susceptibility of the elderly to seasonal or pandemic viral infections? Here, we describe the significance of the work by Guo et al. and summarize the recent advances in the field of NK cell biology and its aging-related clinical relevances [3].

## The paradigm of immune memory in NK cells

NK cells are one of the major effector lymphocytes and have the unique ability to identify infected cells. However, they neither use clonotypic receptors for their development nor use them to mediate their functions. Interactions between MHC Class-I on target cells and the killer immunoglobulin-like receptors in NK cells primarily lead to self-recognition and, therefore, inhibition. Thus, intracellular pathogens that evade CD8^+^ T cells by downregulating MHC Class-I surface expression are vulnerable to NK cell-mediated clearance. In this aspect, NK cells and CD8^+^ T cells represent the innate and adaptive arms of immunity against pathogens, including viruses. This dogma was challenged with the recent discovery of memory-like NK cells [4].

The formation of memory-like NK cells has been extensively investigated in both mice and humans [5]. Murine cytomegalovirus (MCMV) infection is a primary model that helped to establish the phenomenon of memory-like NK cells [6]. Later studies confirmed the existence of memory-like NK cells in mice using antigens from viruses including influenza, vesicular stomatitis virus (VSV), and human immunodeficiency virus type 1 (HIV-1) [7]. Secondary infections with vaccinia and herpes simplex virus type 2 (HSV-2) resulted in the expansion of memory-like NK cells and protection. HCMV infections lead to the generation of NKG2C^+^CD94^+^ NK subset in humans [8]. Recognition of viral proteins such as m157 of MCMV by Ly49H or viral peptides, including UL40-derived VMAPRTLIL, presented by HLA-E recognized by NKG2C/CD94 complex results in the activation and generation of memory-like NK cells. However, the mechanism that sustains their continued proliferation and existence is yet to be fully defined. Emerging evidence indicates that IL-12R signaling may play a role in developing memory NK cells. IL-12-mediated STAT4 activation is necessary for generating MCMV-specific memory NK cells. Treating NK cells with IL-12, IL-18, and low-dose IL-15 induced memory-like, long-lived NK cells. Additional work is warranted to determine the mechanisms and requirements for sustaining and reactivating memory-like NK cells.

NK cells are the major effector lymphocytes that counter HCMV infections, which presents a major threat to immune-compromised patients, including HIV-infected patients or transplant recipients. HCMV is also a significant cause of infection-related congenital defects and abortion. Thus, the work by Guo et al. on HCMV-seropositive and older individuals represent a advancement in our understanding of the potential inflammaging caused by memory-like NK cells [3]. Although memory-like NK cells do not utilize clonotypic receptors, they recognize viral peptide-loaded non-classical MHC Class-I molecule, HLA-E using the NKG2C receptor [9]. The evolutionary adaptation of germline-encoded non-clonotypic NKG2C recognizing HCMV protein derived epitopes on HLA-E parallels the features of adaptive immune cells. However, the complexity of these receptor-ligand interactions does not end with this uniqueness. HLA-E, which is widely expressed on multiple cell types, also presents peptides from leader sequences of classical MHC Class-I or integral peptides of heat shock proteins to another family member, NKG2A, on NK cells. This interaction between HLA-E with self-peptides and NKG2A leads to self-recognition and inhibition of NK cells. Moreover, the affinity of UL40 peptide-loaded HLA-E to NKG2C is much higher than that to NKG2A. Thus, the immune system prefers to activate and generate the memory-like CD52^+^NKG2C^+^CD94^+^ NK cells during viral infections.

While the current finding from Guo et al. [3]. is exciting and sets the stage for future studies, it also raises a number of questions. What are the functions of memory-like NK cells during acute or chronic viral infections? Do they play similar roles against HCMV and SARS-CoV-2 infections? Inflammaging is not a common clinical presentation during HCMV reactivation from latency; however, SARS-CoV-2 infections lead to severe septic-like inflammations in older individuals. Thus, it appears that there may be subsets of memory-like NK cells that predominate depending on the type of infection. We do not know if HLA-E presents any antigenic epitope from SARS-CoV-2 that NKG2C recognizes. Homozygous or heterozygous deletion of the *NKG2C* allele has been associated with the severity of COVID-19 pathology that requires hospitalization and ICU admission [10]. Same genetic alteration in *NKG2C* allele was shown earlier as a risk factor for severe HCMV viremia [11]. Similarly, an HLA-E*0101 allele predominantly present among the European population is associated with hospitalization and hospitalization in the ICU following SARS-CoV-2 infection [10]. These two genetic variants were independent risk factors for severe COVID-19. Thus, the memory-like NK cell subset may play a dual role. Lack of this subset leads to uncontrolled viral growth; however, overactivation may cause them to contribute to inflammaging. In this context, the findings from Guo et al. demonstrate the presence of a higher number of memory-like NK cells, indicatingthat they are an essential component of the innate immune system [3].

## Remaining questions and conclusions

Memory-like NK cells hold promise for novel cell-targeted therapies. However, the following questions need to be addressed in the near future. How do memory-like adaptive NK cells develop? What subset of mature NK cells transitions into memory-like NK cells? What are their contributions to inflammaging? Is there developmental or functional plasticity between memory-like and other terminally differentiated mature subsets of NK cells? There are no clear answers for the longevity of memory-like NK cells, which is a core functional component of immune memory. Given that memory-like NK cells recognize viral antigens, do they need sustained reactivation to maintain the memory characteristics? What are transcriptional and transcriptomic regulations required to establish and maintain the memory-like NK cells? Answers to these questions require a better understanding of their transcriptomic, genetic, and epigenetic alterations. This information will potentially help generate novel NK cell-based therapies, including CAR-NK cells, to formulate better therapeutic approaches against malignancies, chronic viral infections and other age-related illnesses.

## Data Availability

Not applicable.
